# The effects of bisphenol A and its analogs on steroidogenesis in MA-10 Leydig cells and KGN granulosa cells^†^

**DOI:** 10.1093/biolre/ioae165

**Published:** 2024-11-09

**Authors:** Lama Iskandarani, Sabrina Romanelli, Barbara F Hales, Bernard Robaire

**Affiliations:** Department of Pharmacology and Therapeutics, McGill University, Montreal, Quebec, Canada; Department of Pharmacology and Therapeutics, McGill University, Montreal, Quebec, Canada; Department of Pharmacology and Therapeutics, McGill University, Montreal, Quebec, Canada; Department of Pharmacology and Therapeutics, McGill University, Montreal, Quebec, Canada; Department of Obstetrics and Gynecology, McGill University, Montreal, Quebec, Canada

**Keywords:** endocrine-disrupting chemicals, bisphenols, ovarian granulosa cells, murine Leydig cells, steroidogenesis

## Abstract

Bisphenols are a family of chemicals used in the manufacture of consumer products containing polycarbonate plastics and epoxy resins. Studies have shown that exposure to bisphenol A (BPA) may disrupt steroidogenesis and induce adverse effects on male and female reproduction, but little is known about BPA replacements. We determined the effects of six bisphenols on the steroidogenic function of MA-10 Leydig cells and KGN granulosa cells by measuring the levels of progesterone and estradiol produced by these cells as well as the expression of transcripts involved in steroid and cholesterol biosynthesis. MA-10 and KGN cells were exposed for 48 h to one of six bisphenols (0.01–50 μM): BPA, bisphenol F, bisphenol S, bisphenol AF, bisphenol M, or bisphenol TMC, under both basal and dibutyryl cAMP (Bu_2_cAMP)-stimulated conditions. In MA-10 cells, most bisphenols increased the Bu_2_cAMP-stimulated production of progesterone. In KGN cells, there was a general decrease in progesterone production, while estradiol levels were increased following exposure to many bisphenols. Quantitative real-time polymerase chain reaction analyses revealed that all six bisphenols (≥1 μM) upregulated the expression of STAR, a cholesterol transporter, in both cell lines after stimulation. Key transcripts directly involved in steroid and cholesterol biosynthesis were significantly altered in a cell line, chemical, and concentration-dependent manner. Thus, BPA and five of its analogs can disrupt steroid production in two steroidogenic cell lines and alter the levels of transcripts involved in this process. Importantly, BPA replacements do not appear to have fewer effects than BPA.

## Teaser

Bisphenol A and its structural analogs affect progesterone and estradiol production and differentially alter the expression of genes involved in steroidogenesis and cholesterol biosynthesis in MA-10 mouse Leydig cells and KGN human granulosa cells.

## Graphical Abstract

**Graphical Abstract:**
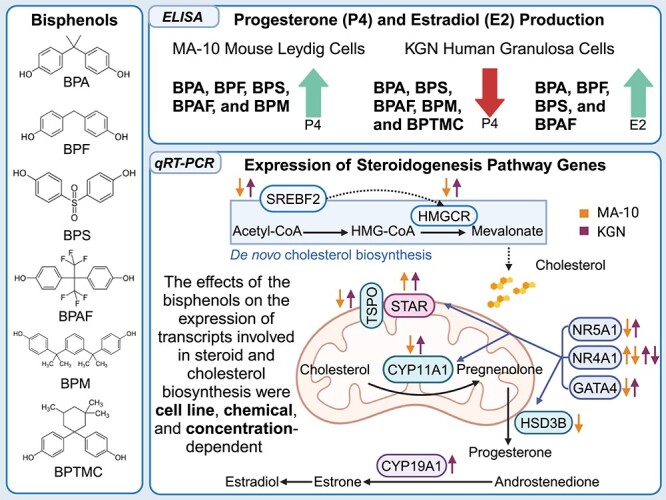

## Introduction

Bisphenol A (BPA) is a high production volume chemical used to produce a wide range of products, including polycarbonate plastics, epoxy resins, and thermal paper receipts [1]. Human exposure to BPA is ubiquitous [2, 3]; exposure occurs mainly through the diet after consumption of BPA-contaminated food or water, but it may also occur via dermal exposure from handling thermal receipts or through dust inhalation [3, 4]. Many studies have associated exposure to BPA with a variety of adverse health effects, including neurodevelopmental disorders, cancer, obesity, and adverse effects on reproduction [5–8]. There is extensive evidence that BPA has the ability to act as an endocrine-disrupting chemical [9, 10].

Because of concerns regarding the adverse health effects associated with exposure to BPA, Canada, the USA, the European Union, and other jurisdictions have implemented regulatory policies that restrict its use in baby bottles and a variety of other products [11–13]. These restrictions, as well as public pressure, have led to a general phase-out of BPA in consumer products. Indeed, there was a statistically significant decrease in the average urinary concentrations of BPA measured in Canadians aged 3 to 79, from 1.2 μg/L in 2009–2011 to 0.68 μg/L in 2018–2019 [14]. As a result of regulations and public pressure, BPA has been replaced in many products, often with other structurally analogous bisphenols, such as bisphenol F (BPF), bisphenol S (BPS), and bisphenol AF (BPAF). Little is known about some of these BPA substitutes, but emerging data suggest that they may have similar or even greater adverse effects than BPA on some endpoints [15]. Even less is known about bisphenol M (BPM) and bisphenol TMC (BPTMC), two other emerging analogs, despite their relatively high detection frequencies in Canadian house dust [16, 17].

Many studies have reported an association between exposure to bisphenols and effects on male and female reproduction. In humans, urinary BPA concentrations were associated with a lower sperm concentration and increased sperm DNA damage [18, 19]. In male rats, exposure to BPA decreased plasma and testicular testosterone levels as well as the expression of several steroidogenic enzymes [20]. Other studies have reported that exposure to BPA induces apoptosis in murine Leydig cells, decreases serum levels of luteinizing hormone (LH) in rats, and downregulates the expression of the LH and follicle-stimulating hormone (FSH) receptors in zebrafish [21–23]. Exposure to 10 μM BPA decreased testosterone secretion in fetal rat testis organ cultures [24]. Exposure to BPA, BPF, or BPS altered steroidogenesis in MA-10 Leydig cells [25]. In women undergoing IVF, increases in urinary BPA concentrations were associated with a decrease in the numbers of antral follicles and of oocytes that were retrieved [26, 27]. Bisphenol A may also adversely affect fertility by impacting on folliculogenesis, steroidogenesis, oocyte quantity and quality, implantation, and pregnancy outcomes [28]. In rat granulosa cells, exposure to BPA decreased estradiol production as well as the mRNA expression of aromatase, increased the levels of progesterone, and increased the expression levels of steroidogenic acute regulatory protein (*Star*) and cytochrome P450 family 11 subfamily A member 1 (cholesterol side-chain cleavage enzyme, *Cyp11a1*) [29]. In ovine granulosa cells, exposure to BPA or BPS (10 or 50 μM) decreased progesterone secretion, while estradiol secretion was only reduced by BPS [30]. In female rats, exposure to BPA reduced serum estradiol concentrations, as well as aromatase and STAR protein levels [31]. There is little information on the effects of some BPA analogs, especially BPM and BPTMC.

The molecular mechanisms by which exposure to bisphenols may interfere with the production of sex hormones, and thus adversely affect male and female fertility, are not clear. The goal of this study was to compare and contrast the effects of BPA and five structural analogs on progesterone and estradiol production in MA-10 mouse tumor Leydig cells and KGN human ovarian granulosa cells and to determine how key transcripts that are directly involved in the steroidogenic pathway and cholesterol biosynthesis are affected.

## Materials and methods

### Chemicals and reagents

The list of bisphenols tested in this study is provided in Table 1. These are 4,4′-(propane-2,2-diyl)diphenol (bisphenol A; BPA), bis(4-hydroxyphenyl)methane (bisphenol F; BPF), bis(4-hydroxyphenyl) sulfone (bisphenol S; BPS), 4-[1,1,1,3,3,3-hexafluoro-2-(4-hydroxyphenyl)propan-2-yl]phenol (bisphenol AF; BPAF), 1,3-bis[2-(4-hydroxyphenyl)-2-propyl]benzene (bisphenol M; BPM), and 1,1-bis(4-hydroyphenyl)-3,3,5-trimethyl-cyclohexane (bisphenol TMC; BPTMC). Bisphenol M and BPTMC were purchased from Toronto Research Chemicals (Toronto, Ontario, Canada). All bisphenols were dissolved in dimethyl sulfoxide (DMSO) from MilliporeSigma Canada Ltd. (Oakville, ON, Canada).

**Table 1:** Names, CAS registry numbers, chemical structures, and sources of the six bisphenols studied

**Compound name (acronym)**	**Systematic name**	**CAS**	**Chemical structure**	**Supplier (purity)**
Bisphenol A (BPA)	4,4′-(propane-2,2-diyl)diphenol	80-05-7	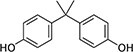	Sigma-Aldrich (98.1%)
Bisphenol F (BPF)	bis(4-hydroxyphenyl)methane	620-92-8	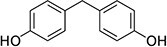	Sigma-Aldrich (99.7%)
Bisphenol S (BPS)	bis(4-hydroxyphenyl) sulfone	80-09-1	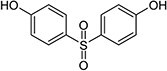	Sigma-Aldrich (99.9%)
Bisphenol AF (BPAF)	4-[1,1,1,3,3,3-hexafluoro-2-(4-hydroxyphenyl)propan-2-yl]phenol	1478-61-1	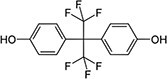	3B Pharmachem International Co. Ltd. (98.0%)
Bisphenol M (BPM)	1,3-bis[2-(4-hydroxyphenyl)-2-propyl]benzene	13595-25-0	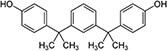	Toronto Research Chemicals
Bisphenol TMC (BPTMC)	1,1-bis(4-hydroyphenyl)-3,3,5-trimethyl-cyclohexane	129188-99-4	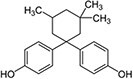	Toronto Research Chemicals

DMEM/F-12 culture medium (Dulbecco Modified Eagle Medium/Nutrient Mixture F-12 with L-glutamine, 15 mM HEPES, and no phenol red) and collagen I rat tail (3 mg/mL) from Gibco were purchased from ThermoFisher Scientific (Burlington, ON, Canada). Charcoal-stripped fetal bovine serum, charcoal-stripped heat-inactivated horse serum, HEPES, 100× penicillin–streptomycin, 1× phosphate buffered saline, and trypsin–EDTA (2.5% trypsin with 22.1 mM EDTA, no phenol red) were obtained from Wisent Inc. (St-Jean-Baptiste, QC, Canada). N6,2′-O-dibutyryladenosine-3′,5′-cyclic monophosphate sodium salt (Bu_2_cAMP, a cell-permeable analog of cAMP; HPLC ≥97%), trypsin–EDTA (0.5% trypsin with 0.2% EDTA, no phenol red), and DMSO were from MilliporeSigma Canada Ltd. Hoechst 33342 from Invitrogen Life Sciences was purchased from ThermoFisher Scientific. Progesterone enzyme-linked immunosorbent assay (ELISA) kits (catalog # RE52231) and estradiol ELISA kits (catalog # RE52041) from IBL International GmbH were purchased from Cedarlane Laboratories (Burlington, ON, Canada).

### Cell cultures

MA-10 Leydig cells were a gift from Dr. Mario Ascoli (University of Iowa) [32]. KGN granulosa cells were a gift from Dr. Christopher Price and Dr. Bruce Murphy (Université de Montréal). Authorization to use KGN cells was provided by Dr. Toshihiko Yanase (Fukuoka University, Japan) [33]. MA-10 cells were cultured in phenol red–free DMEM/F-12 medium supplemented with 5% charcoal-stripped fetal bovine serum, 2.5% charcoal-stripped horse serum, 20 mM HEPES, and 0.5% penicillin–streptomycin. KGN cells were cultured in phenol red–free DMEM/F-12 medium supplemented with 10% charcoal-stripped fetal bovine serum and 0.5% penicillin–streptomycin. Both cell lines were cultured in 75-cm^2^ or 175-cm^2^ Corning virgin polystyrene cell culture flasks, and kept in a humidified atmosphere at 37°C with 5% CO_2_. The culture medium was renewed every 2 to 3 days.

### High-content imaging

To determine cell counts, MA-10 cells (10,000 cells/well) and KGN cells (60,000 cells/well) were cultured in 96-well sterile black opaque plates (PhenoPlate by Revvity, catalog # 6055300; Woodbridge, ON, Canada). The plates that were used for KGN cells were coated with 0.2% collagen I rat tail to facilitate the adhesion of the cells to the plate. The stock solutions for each bisphenol were prepared in DMSO; the compounds were then diluted to the desired concentrations in complete culture medium. Dimethyl sulfoxide was maintained at 0.5% in the vehicle control and all bisphenol concentrations tested. The concentrations tested in the present study were chosen to avoid overt cytotoxicity, while still encompassing environmentally relevant exposures [14, 17, 34]. Cells were seeded in the plates and allowed to attach for 24 h prior to exposure to a bisphenol. MA-10 cells were exposed to BPA, BPF, BPS, or BPAF at concentrations of 1, 5, 10, or 20 μM; to BPM at concentrations of 0.01, 0.1, 1, or 5 μM; and to BPTMC at concentrations of 0.1, 1, 5, or 10 μM. KGN cells were exposed to BPA, BPF, BPS, or BPAF at concentrations of 5, 10, 20, or 50 μM, and to BPM or BPTMC at concentrations of 1, 5, 10, or 20 μM.

MA-10 cells were incubated with 200 μL of complete medium containing 0.5% DMSO (vehicle control) or the different concentrations of each bisphenol for 48 h. Following the chemical exposure, medium was removed and the cells were washed twice with serum-free medium. To determine basal progesterone production, MA-10 cells were cultured in serum-free medium for 2 h; the culture medium was collected and stored at −80°C for subsequent progesterone hormone measurements. To assess stimulated progesterone production, MA-10 cells were cultured in serum-free medium containing 1 mM Bu_2_cAMP for 2 h. Following the collection of the culture medium, the cells were stained for 30 min with Hoechst 33342 fluorescent dye diluted in serum-free medium. KGN cells were incubated with 200 μL of complete medium containing 0.5% DMSO (vehicle control) or the different concentrations of each bisphenol to assess basal (complete medium) and stimulated (complete medium containing 1 mM Bu_2_cAMP) progesterone production for 48 h. In order to measure estradiol production, KGN cells were additionally supplemented with 10 μM androstenedione (MilliporeSigma Canada Ltd.). Following the chemical exposure, the conditioned media in duplicate wells were pooled and stored at −80°C, after which the cells were stained with Hoechst 33342 for 30 min.

Total cell counts per well (Supplementary Figure S1) were assessed after staining with Hoechst 33342 dye using the PerkinElmer Operetta High-Content Imaging System and analyzed with the Columbus Image Data Storage and Analysis System (Revvity).

### Progesterone and estradiol assays

The progesterone concentrations produced by MA-10 or KGN cells and the estradiol concentrations produced by KGN cells were assessed using ELISA kits following the manufacturer’s protocol; samples were measured in duplicate. Absorbance in the wells was read at 450-nm wavelength with a SpectraMax Plus 384 plate reader (Molecular Devices). The analytical sensitivities of the progesterone and estradiol ELISA were 0.045 ng/mL and 10.6 pg/mL, respectively. For MA-10 ELISA plates, the mean intra-assay variation was 3.2% and the mean inter-assay variation was 20.9%. For KGN ELISA plates, the mean intra-assay and inter-assay variation was 8.8% and 9.2% for the progesterone assays and 8.0% and 11.2% for the estradiol assays.

### RNA extraction

The concentrations chosen for quantitative real-time polymerase chain reaction (qRT-PCR) experiments were 1 and 5 μM for MA-10 cells and 5 and 20 μM for KGN cells. Based on previous studies, cell numbers were maintained at a minimum of 70% of control at these concentrations [17, 35]. MA-10 cells (75,000 cells/well) were seeded in 6-well plates for 24 h, then exposed to control, 1, or 5 μM of each bisphenol for 48 h, followed by a 2-h stimulation with serum-free medium containing Bu_2_cAMP. KGN cells (360,000 cells/well) were seeded in collagen-coated 24-well plates for 24 h, then exposed to control, 5, or 20 μM of each bisphenol, with (stimulated) or without (basal) complete medium containing 1 mM Bu_2_cAMP for 48 h.

Cells were lysed directly in the plates and RNA was extracted using RNeasy Plus Mini Kits (Qiagen; Toronto, ON, Canada) according to the manufacturer’s protocol with slight modifications. For KGN cells, the cell lysate was pooled from duplicate wells prior to RNA extraction in a total of 350 μL of Buffer RLT Plus. All lysates were passed through QIAshredder spin columns to ensure homogenization; lysates were centrifuged for 30 s at a speed of 13,523×*g*. The extracted RNA was eluted twice in 30 μL of RNase-free water. The concentration and purity of the isolated RNA were assessed using a NanoDrop 2000 spectrophotometer (ThermoFisher Scientific).

### Quantitative real-time polymerase chain reaction

RNA samples were diluted to a working concentration of 2 ng/μL and transcripts were quantified using a Power SYBR Green RNA-to-C_T_-1-Step Kit from Applied Biosystems (ThermoFisher Scientific), and the StepOne Real-Time PCR System or the Viia7 Real-Time PCR System. Each 20-μL reaction was run in triplicate and consisted of 10 μL SYBR Green Master Mix, 2.84 μL RNase–DNase-free water, 2 μL of primer, 0.16 μL of Reverse Transcriptase Mix, and 5 μL of the RNA sample (or sterile water for the negative controls). The following conditions were used for PCR: 48°C for 30 min, 95°C for 10 min (hold stage), followed by 40 cycles of 95°C for 15 s, 55°C for 30 s, and 72°C for 30 s (PCR stage). Finally, the melt curve stage consisted of 95°C for 15 s, 60°C for 15 s, and 95°C for 15 s.

Primers were purchased from QuantiTect Primer Assays (Qiagen), with the exception of *CYP19A1*, which was obtained from Integrated DNA Technologies (Coralville, IA, USA). The primers for MA-10 cells were steroidogenic acute regulatory protein (*Star*; QT00165977); translocator protein (*Tspo*; QT01750217); cholesterol side-chain cleavage enzyme (*Cyp11a1*; QT00161091); hydroxy-delta-5-steroid dehydrogenase (*Hsd3b1*; QT00266231); 3-hydroxy-3-methylglutaryl-Coenzyme A reductase (*Hmgcr*; QT01037848); sterol regulatory element binding factor 2 (*Srebf2*; QT01045870); nuclear receptor subfamily 5, group A, member 1 (*Nr5a1*; QT00120169); nuclear receptor subfamily 4, group A, member 1 (*Nr4a1*; QT00101017); and GATA binding protein 4 (*Gata4*; QT00155400). Hypoxanthine guanine phosphoribosyltransferase (*Hprt*; QT00166768) was used as a housekeeping gene.

The primers for KGN cells were steroidogenic acute regulatory protein (*STAR*; QT00091959); translocator protein (*TSPO*; QT00997731); cholesterol side-chain cleavage enzyme (*CYP11A1*; QT00040117); hydroxy-delta-5-steroid dehydrogenase (*HSD3B2*; QT00000490); 3-hydroxy-3-methylglutaryl-CoA reductase (*HMGCR*; QT00004081); sterol regulatory element binding transcription factor 2 (*SREBF2*; QT00052052); nuclear receptor subfamily 5, group A, member 1 (*NR5A1*; QT00088018); nuclear receptor subfamily 4, group A, member 1 (*NR4A1*; QT00095515); and GATA binding protein 4 (*GATA4*; QT00031997). The cytochrome P450, family 19, subfamily A, member 1 (*CYP19A1*) transcript was custom designed with 5′-TGC AAA GCA CCC TAA TGT TG-3′ as the forward primer and 5′-CAT GAC CAA GTC CAC GAC AG-3′ as the reverse primer, prepared in 1× Tris–EDTA buffer at a concentration of 12.5 μM. Glyceraldehyde-3-phosphate dehydrogenase (*GAPDH*; QT00079247) was used as a housekeeping gene.

A reference sample (RNA from normally cultured MA-10 or KGN cells) was run on each plate for calibration, using the ΔΔC_T_ method [36, 37]. Polymerase chain reaction data were analyzed using the StepOnePlus Software (version 2.3) or the QuantStudio Real-Time PCR Software (version 1.3). Triplicates were manually checked for outliers (deviation of >0.2 C_T_ value from the other two values), which were excluded. Expression levels were normalized to the housekeeping gene transcripts. Relative quantification results were plotted as fold changes compared to the basal controls (2^−ΔΔCT^).

### Statistical analyses

Each experiment had technical duplicates; experiments were repeated five to eight times for MA-10 cells and five to six times for KGN cells (biological replicates). Data were analyzed using GraphPad Prism 8 software (version 8.3.0). Progesterone and estradiol concentrations were compared using two-way repeated measures analysis of variance (ANOVA) followed by Dunnett's test, and mRNA expression levels were compared using two-way ANOVA followed by Dunnett's test to determine significant differences from the controls in each condition (basal and stimulated). Cell counts were compared using Holm–Bonferroni corrected one-sample *t*-tests. *P* <0.05 was considered statistically significant.

## Results

### Effects of exposure to bisphenols on cell counts

Total cell counts were assessed to determine the cytotoxicity of each bisphenol (Supplementary Figure S1). In MA-10 cells, cell survival was ≥70% under basal and Bu_2_cAMP-stimulated conditions for the highest concentrations tested: 20 μM BPA and BPAF, 5 μM BPM, and 10 μM BPTMC. In KGN cells, none of the bisphenols caused more than an 8% decrease in cell counts from control levels at the concentrations tested (Supplementary Figure S1B).

### Effects of exposure to bisphenols on progesterone secretion in MA-10 and KGN cells

**Figure 1:**
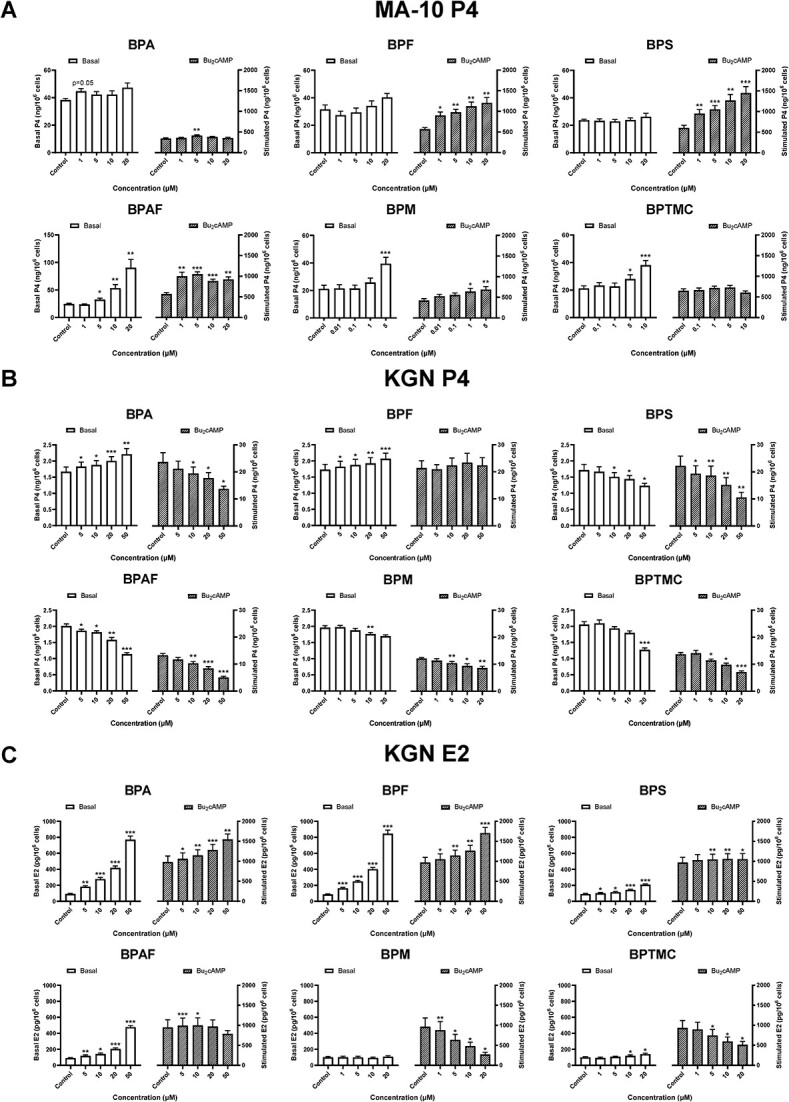
Effects of bisphenols on basal and dibutyryl-cAMP (Bu_2_cAMP)-stimulated progesterone and estradiol levels. (A) MA-10 cells were treated for 48 h, followed by a 2-h incubation with or without Bu_2_cAMP. (B) KGN cells were treated for 48 h in the absence or presence of Bu_2_cAMP. (C) KGN cells were treated for 48 h in the absence or presence of Bu_2_cAMP and were additionally supplemented with 10 μM androstenedione. Supernatant was collected and progesterone (P4) or estradiol (E2) was measured in the medium. All cells were stained with Hoechst 33342 and counted by high-content imaging for normalization (progesterone levels are displayed as ng or pg per 10^6^ cells ± SEM). Two-way repeated measures ANOVA followed by Dunnett test was performed to determine significant differences from the controls in each condition; ^*^P < 0.05, ^**^P < 0.01, ^***^P < 0.001, *N* = 8 (MA-10) and *N* = 6 (KGN).

Progesterone (P4) was measured in the culture medium of MA-10 cells exposed to BPA or its analogs for 48 h to assess their impact on steroidogenic capabilities (Figure 1A). The average production of P4 by MA-10 cells was 26.7 ± 2.8 ng/10^6^ cells under basal conditions. Exposure to BPA had a minimal effect (1 μM BPA: *P* = 0.05); neither BPF nor BPS affected basal P4 production; in contrast, exposure to BPAF, BPM, or BPTMC significantly increased basal P4 secretion. Bisphenol AF (20 μM) induced a ≥3-fold increase in P4 levels; BPM (5 μM) and BPTMC (10 μM) induced increases of 91% and 81%, respectively. In MA-10 cells, the average production of P4 after Bu_2_cAMP stimulation in control samples was 525.6 ± 48.2 ng/10^6^ cells, an almost 20-fold increase in comparison with basal P4 production (Figure 1A). Progesterone production after Bu_2_cAMP stimulation levels was further increased by exposure to all of the bisphenols studied with the exception of BPTMC. A further increase in P4 production after Bu_2_cAMP stimulation was observed for BPA (5 μM), BPF, BPS, and BPAF (at concentrations ranging from 1 to 20 μM) and BPM (1 and 5 μM). Overall, only BPAF, BPM, and BPTMC altered the basal production of P4 in MA-10 cells; every bisphenol except BPTMC affected the response of these cells to Bu_2_cAMP stimulation.

Under basal conditions, the average production of P4 by KGN cells in 48 h was 1.9 ± 0.07 ng/10^6^ cells (Figure 1B); after stimulation, P4 production was increased ~9-fold, to an average of 17.7 ± 2.1 ng/10^6^ cells. Exposure to BPA or BPF increased basal P4 secretion in a concentration-dependent manner; in contrast, a decrease in P4 secretion of up to 50% was observed after BPS, BPAF, BPM, or BPTMC exposure. After supplementation with Bu_2_cAMP, a decrease in the secretion of P4 was observed after exposure to five of the six bisphenols; the largest decrease (~62%) was observed after exposure to 50 μM BPAF. Notably, BPF did not affect Bu_2_cAMP-stimulated P4 secretion.

### Effects of exposure to bisphenols on estradiol secretion in KGN cells

In order to further assess the impact of BPA and its analogs on the steroidogenic capabilities of KGN cells, estradiol (E2) was measured in the culture medium after 48 h of exposure (Figure 1C). The average production of E2 by KGN cells was 92.8 ± 2.0 pg/10^6^ under basal conditions. Bisphenol A, BPF, BPS, BPAF, and BPTMC increased the basal levels of estradiol, with BPA and BPF causing the most drastic effects. Bisphenol M did not significantly alter the basal concentration of estradiol. After Bu_2_cAMP stimulation, the average production of E2 in control samples was 961.3 ± 7.1 pg/10^6^ cells, an almost 11-fold increase in comparison with basal E2 production (Figure 1C). Exposure to BPA, BPF, BPS, and BPAF further increased the levels of estradiol, while BPM and BPTMC caused a decrease in estradiol production. Thus, BPA and its analogs had differential effects on the synthesis of P4 and E2 in KGN cells and their ability to respond to Bu_2_cAMP stimulation. A graphical representation of these data is shown in Supplementary Figure S2.

### Effects of exposure to bisphenols on the expression of cholesterol transporters in MA-10 and KGN cells

**Figure 2:**
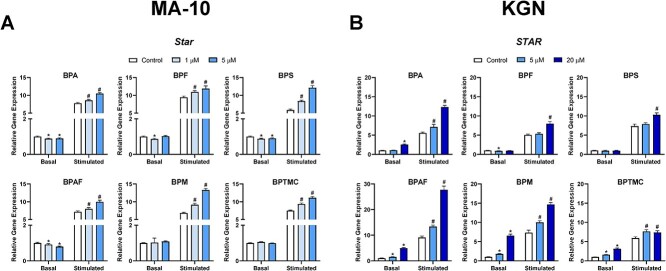
Effects of bisphenols on the mRNA expression of steroidogenic acute regulatory protein (STAR), a cholesterol transporter. (A) MA-10 cells were treated for 48 h with control, 1, or 5 μM of each bisphenol, followed by a 2-h incubation with or without Bu_2_cAMP. (B) KGN cells were treated for 48 h with control, 5, or 20 μM of each bisphenol, in the absence or presence of Bu_2_cAMP. Bar graphs show the effects of the six bisphenols on the relative expression of (A) *Star* and (B) *STAR*. Data represent means ± 95% confidence interval (CI). qRT-PCR expression was normalized to *Hprt* (MA-10 cells) or *GAPDH* (KGN cells). Two-way ANOVA with Dunnett test was performed; ^*^P < 0.05 compared to basal control, ^#^*P* < 0.05 compared to stimulated control, *N* = 5–7 (MA-10) and *N* = 5 (KGN).

To elucidate the mechanism(s) by which exposure to bisphenols affects the production of steroid hormones by MA-10 and KGN cells, we determined whether these chemicals altered the expression of two key transcripts involved in the transportation of cholesterol from the outer to the inner mitochondrial membrane, steroidogenic acute regulatory protein (*Star*) and translocator protein (*Tspo*) (Figure 2, Supplementary Figure S3). In MA-10 cells, under basal conditions, exposure to BPA, BPF, BPS, or BPAF induced small but significant decreases in *Star* expression compared to control; BPM and BPTMC had no significant effect on its expression (Figure 2A). Bu_2_cAMP stimulation drastically increased the expression of *Star*, and this was further upregulated by exposure to all six bisphenols. In KGN cells, basal *STAR* expression was increased after exposure to BPA, BPAF, BPM, or BPTMC, not affected by BPS, and decreased after exposure to BPF (Figure 2B). *STAR* expression in KGN cells was also stimulated by Bu_2_cAMP and further increased by exposure to all the bisphenols tested. Exposure to 20 μM BPAF upregulated *STAR* expression 3-fold above the stimulated control.

In MA-10 cells, exposure to bisphenols downregulated the expression of *Tspo* under both basal and stimulated conditions (Supplementary Figure S3A). The minimal impact of exposure to bisphenols on *Tspo* expression may indicate that the changes observed in P4 production are due to the role of *Star* in the transport of cholesterol into the mitochondria, rather than an effect on *Tspo*. In KGN cells, stimulation alone decreased the expression of *TSPO* (Supplementary Figure S3B).

### Effects of exposure to bisphenols on transcripts involved in steroidogenesis

**Figure 3:**
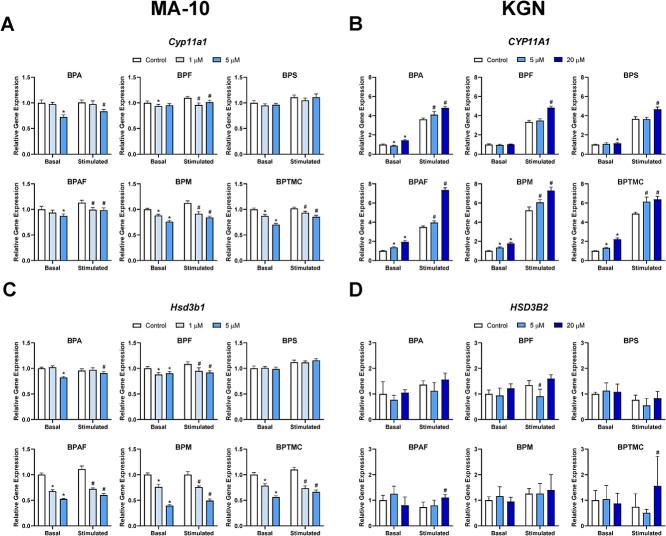
Effects of bisphenols on the mRNA expression levels of two key steroidogenic enzymes. MA-10 cells (left) were treated for 48 h with control, 1, or 5 μM of each bisphenol, followed by a 2-h incubation with or without Bu_2_cAMP. KGN cells (right) were treated for 48 h with control, 5, or 20 μM of each bisphenol, in the absence or presence of Bu_2_cAMP. Bar graphs show the effects of the six bisphenols on the relative expression of (A) *Cyp11a1*, (B) *CYP11A1*, (C) *Hsd3b1*, and (D) *HSD3B2*. Data represent means ± 95% CI. qRT-PCR expression was normalized to *Hprt* (MA-10 cells) or *GAPDH* (KGN cells). Two-way ANOVA with Dunnett test was performed; ^*^P < 0.05 compared to basal control, *^#^P* < 0.05 compared to stimulated control, *N* = 5–7 (MA-10) and *N* = 5 (KGN).

The effects of exposure to bisphenols on the expression of cholesterol side-chain cleavage enzyme (CYP11A1) and hydroxy-delta-5-steroid dehydrogenase (HSD3B) were assessed in both MA-10 and KGN cells (Figure 3). Under basal conditions, *Cyp11a1* expression was downregulated in MA-10 cells by all bisphenols except for BPS (Figure 3A). Bisphenol A and BPAF decreased expression levels only in cells exposed to a concentration of 5 μM (BPA: 28%; BPAF: 13%); BPF induced downregulation (6%) only in cells exposed to 1 μM. Bisphenol M and BPTMC decreased relative levels of *Cyp11a1* at both of the concentrations tested. Stimulation by Bu_2_cAMP alone did not have a major impact on *Cyp11a1* expression in MA-10 cells. Thus, all the bisphenols except for BPS significantly decreased *Cyp11a1* expression, with BPF and BPAF causing the smallest (~12%) and BPM causing the largest (25%) decrease (Figure 3A).

In KGN cells, all the bisphenols except for BPF increased basal *CYP11A1* expression (Figure 3B). Exposure to 5 μM BPA caused a slight downregulation of expression levels, which then switched to an upregulation in cells exposed to 20 μM. Exposure to 20 μM BPA or BPS induced modest changes, with increases of expression to 1.4-fold and 1.1-fold of basal control, respectively. Exposure to 20 μM BPAF, BPM, or BPTMC led to larger increases in *CYP11A1* expression levels, reaching 1.9-, 1.8-, and 2.2-fold of control levels, respectively. Stimulation with Bu_2_cAMP led to a large upregulation in *CYP11A1* expression levels in KGN cells, ranging from 3.3- to 5.2-fold of the basal control (Figure 3B). After stimulation, all six bisphenols upregulated *CYP11A1* after exposure to either concentration tested (BPA, BPAF, BPM, and BPTMC) or only after exposure to 20 μM (BPF and BPS). Exposure to 20 μM BPAF increased *CYP11A1* expression by 110% compared to the stimulated control.

Hydroxy-delta-5-steroid dehydrogenase (HSD3B) is a key enzyme in the synthesis of all bioactive steroids, including progesterone and estradiol. Since the expression of HSD3B isozymes is tissue specific, we assessed *Hsd3b1* transcript levels in MA-10 cells and *HSD3B2* levels in KGN cells (Figure 3). All the bisphenols, except for BPS, altered the expression of *Hsd3b1* (Figure 3C). Under basal conditions, BPA (5 μM) and BPF (1 or 5 μM) caused a modest decrease in *Hsd3b1*. In contrast, exposure to 5 μM BPAF, BPM, or BPTMC downregulated this transcript by 48%, 61%, and 44%, respectively. Stimulation did not greatly alter the expression levels of *Hsd3b1* in MA-10 cells. Bisphenol A and BPF induced a small (5–15%) decrease in *Hsd3b1* expression in the presence of Bu_2_cAMP. The downregulation of *Hsd3b1* after exposure to 5 μM BPAF, BPM, or BPTMC (46%, 51%, and 40%) under stimulated conditions was in the same order of magnitude. In KGN cells, *HSD3B2* expression levels were similar under basal and stimulated conditions (Figure 3D). Only 5 μM BPF downregulated Bu_2_cAMP-induced expression of *HSD3B2*. Treatment with 20 μM BPAF and BPTMC increased the expression of this transcript after stimulation (Figure 3D).

The relative expression of cytochrome P450 family 19 subfamily A member 1 (aromatase, *CYP19A1*), the enzyme responsible for the aromatization of androgens to estrogens, was assessed in KGN cells (Supplementary Figure S3C). Exposure to 5 μM BPA decreased the levels of *CYP19A1* transcripts to one-third of control in the absence of stimulation; in contrast, 5 μM BPF, 20 μM BPS, 20 μM BPAF, 5 μM BPM, or 20 μM BPTMC upregulated basal *CYP19A1* expression. Stimulation with Bu_2_cAMP induced a major increase in *CYP19A1* expression. Exposure to 20 μM of four of the five BPA analogs tested further increased *CYP19A1* levels by 95% (BPA), 123% (BPF), 39% (BPS), 98% (BPAF), or 42% (BPTMC) compared to the stimulated controls, while exposure to BPM decreased *CYP19A1* expression (Supplementary Figure S3C).

### Effects of exposure to bisphenols on upstream regulators of steroidogenesis

**Figure 4:**
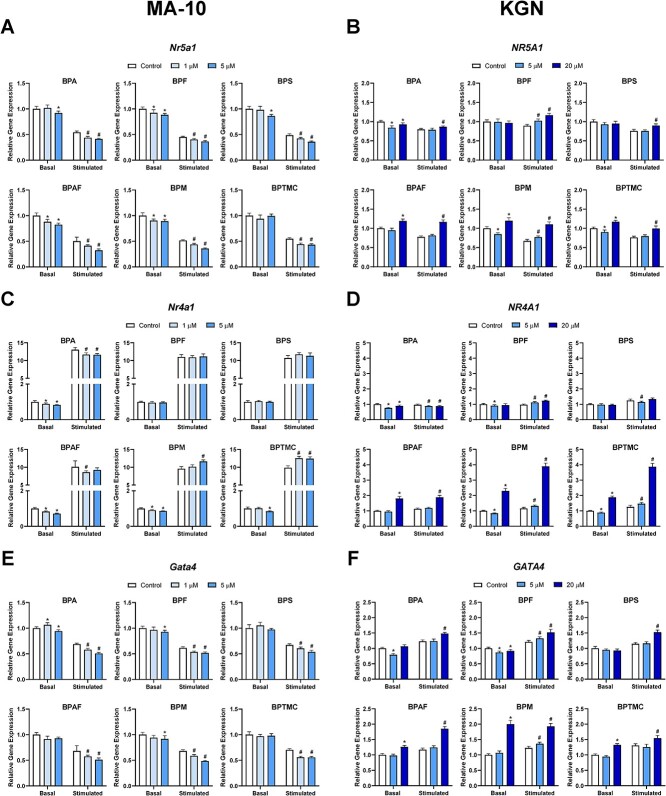
Effects of bisphenols on the mRNA expression levels of the upstream regulators of STAR. MA-10 cells (left) were treated for 48 h with control, 1, or 5 μM of each bisphenol, followed by a 2-h incubation with or without Bu_2_cAMP. KGN cells (right) were treated for 48 h with control, 5, or 20 μM of each bisphenol, in the absence or presence of Bu_2_cAMP. Bar graphs show the effects of the six bisphenols on the relative expression of (A) *Nr5a1*, (B) *NR5A1*, (C) *Nr4a1*, (D) *NR4A1*, (E) *Gata4*, and (F) *GATA4*. Data represent means ± 95% CI. qRT-PCR expression was normalized to *Hprt* (MA-10) or *GAPDH* (KGN). Two-way ANOVA with Dunnett test was performed; ^*^P < 0.05 compared to basal control, ^#^*P* < 0.05 compared to stimulated control, *N* = 5–7 (MA-10) and *N* = 5 (KGN).

Since bisphenols induced changes in the expression of transcripts involved in steroidogenesis in both MA-10 and KGN cells, we determined whether they also affected the expression of three upstream regulators, steroidogenic factor 1/NR5A1 (MA-10: *Nr5a1*; KGN: *NR5A1*), NUR77 (MA-10: *Nr4a1*; KGN: *NR4A1*), and GATA binding protein 4 (MA-10: *Gata4*; KGN: *GATA4*) (Figure 4). All bisphenols, with the exception of BPTMC, decreased the relative expression of *Nr5a1* in MA-10 cells under basal conditions (Figure 4A); the decrease observed ranged from 8% (BPA) to 18% (BPAF). Stimulation with Bu_2_cAMP downregulated the relative expression of *Nr5a1* to approximately half of the basal level; this expression was further downregulated after treatment with all six bisphenols. In KGN cells under basal conditions, exposure to BPA (5 or 20 μM) downregulated the expression of *NR5A1* (Figure 4B). Bisphenol F and BPS had no effect, whereas BPM and BPTMC induced biphasic changes: *NR5A1* expression was downregulated by exposure to 5 μM and upregulated by 20 μM. *NR5A1* expression was upregulated by all bisphenols (5 or 20 μM) after Bu_2_cAMP stimulation (Figure 4B).

In MA-10 cells, stimulation alone drastically upregulated *Nr4a1* expression, which reached 9.5- to 13-fold of basal levels (Figure 4C). Bisphenol F and BPS had no effect on the basal expression of *Nr4a1*, whereas it was downregulated by exposure to BPA, BPAF, BPM, or BPTMC. Exposure to 5 μM decreased relative gene expression of this transcript by 17% (BPA), 28% (BPAF), 14% (BPM), or 15% (BPTMC). Bisphenol F and BPS also had no effect on the Bu_2_cAMP-induced expression of *Nr4a1*. Bisphenol A (1 or 5 μM) and BPAF (1 μM) downregulated *Nr4a1* expression after stimulation, whereas BPM (5 μM) and BPTMC (1 and 5 μM) upregulated it.

In KGN cells, Bu_2_cAMP stimulation did not drastically alter *NR4A1* expression (Figure 4D). Both concentrations of BPA downregulated *NR4A1* expression under basal and stimulated conditions. Exposure to 5 μM BPF reduced basal *NR4A1* expression while 5 and 20 μM upregulated *NR4A1* expression after stimulation. Exposure to BPS (5 μM) downregulated *NR4A1* expression only after stimulation. Exposure to 20 μM BPAF increased basal *NR4A1* expression to 1.8-fold of control; after stimulation, BPAF (20 μM) expression was increased by 66%. Under basal conditions, BPM and BPTMC induced biphasic changes: exposure to 5 μM downregulated and 20 μM upregulated *NR4A1*. Under stimulated conditions, BPM and BPTMC (5 μM) increased the expression of this transcript; exposure to 20 μM of either chemical induced a >200% increase in the relative expression of *NR4A1* (Figure 4D).

GATA4 is essential during the development of several organs, including the testis and the ovary [38, 39]. *Gata4* expression was downregulated in MA-10 cells exposed to 5 μM BPA, BPF, or BPM under basal conditions; BPS, BPAF, and BPTMC had no significant effects on relative gene expression of *Gata4* (Figure 4E). The addition of Bu_2_cAMP decreased *Gata4* expression; transcript levels were further decreased after exposure to 1 or 5 μM of all six bisphenols. In KGN cells under basal conditions, BPA (5 μM) and BPF (5 or 20 μM) downregulated *GATA4* expression, while BPS had no significant effect (Figure 4F). Exposure to BPAF, BPM, or BPTMC (20 μM) upregulated relative gene expression to 1.3- to 2-fold of basal control. Bu_2_cAMP alone slightly upregulated *GATA4* expression. All six bisphenols (5 or 20 μM) upregulated *GATA4* expression under Bu_2_cAMP-stimulated conditions (Figure 4F).

### Effects of exposure to bisphenols on transcripts involved in cholesterol biosynthesis

**Figure 5:**
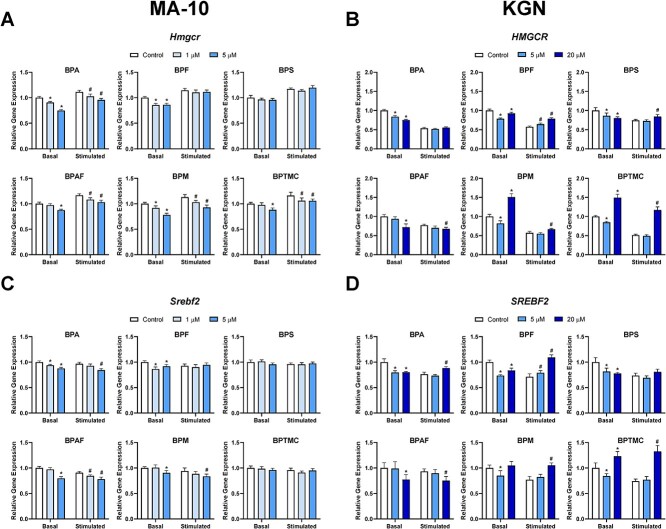
Effects of bisphenols on the mRNA expression levels of transcripts involved in cholesterol biosynthesis. MA-10 cells (left) were treated for 48 h with control, 1, or 5 μM of each bisphenol, followed by a 2-h incubation with or without Bu_2_cAMP. KGN cells (right) were treated for 48 h with control, 5, or 20 μM of each bisphenol, in the absence or presence of Bu_2_cAMP. Bar graphs show the effects of the six bisphenols on the relative expression of (A) *Hmgcr*, (B) *HMGCR*, (C) *Srebf2*, and (D) *SREBF2*. Data represent means ± 95% CI. qRT-PCR expression was normalized to *Hprt* (MA-10) or *GAPDH* (KGN). Two-way ANOVA with Dunnett test was performed; ^*^P < 0.05 compared to basal control, ^#^*P* < 0.05 compared to stimulated control, *N* = 5–7 (MA-10) and *N* = 5 (KGN).

Since STAR mRNA levels were altered after cells were exposed to BPA and its analogs, we determined whether the transcripts upstream of STAR were impacted, namely, those involved in the de novo cholesterol biosynthesis pathway. Therefore, we assessed the mRNA expression levels of HMGCR, the rate-limiting enzyme of the cholesterol biosynthesis pathway, as well as SREBF2, the upstream regulator of this pathway (Figure 5). In MA-10 cells, Bu_2_cAMP induced a small increase in *Hmgcr* expression (Figure 5A). Under basal conditions, both test concentrations of BPA, BPF, or BPM significantly decreased *Hmgcr* expression. Exposure to 5 μM BPAF or BPTMC also downregulated *Hmgcr* expression, while BPS had no significant effect. In the presence of Bu_2_cAMP, BPA, BPAF, BPM, and BPTMC decreased the relative gene expression in a concentration-dependent manner; BPF and BPS had no significant effects (Figure 5A).

In KGN cells, basal *HMGCR* levels were decreased to 0.75-fold of control after treatment with 20 μM BPA (Figure 5B). Similar changes were observed after exposure to 5 μM BPF (0.78-fold), 20 μM BPS (0.80-fold), or 20 μM BPAF (0.72-fold). Bisphenol M and BPTMC once again had biphasic effects, with 5 μM exposure downregulating expression and 20 μM exposure upregulating it. Stimulation alone reduced *HMGCR* levels, but the bisphenols had more varied effects. Bisphenol A had no significant effect, and BPF, at both concentrations tested, upregulated *HMGCR* expression. Bisphenol S, BPM, and BPTMC only increased *HMGCR* levels at the higher concentration tested; BPTMC increased gene expression by 128% compared to its stimulated control. Bisphenol AF induced a downregulation of gene expression at 20 μM (Figure 5B).

**Figure 6:**
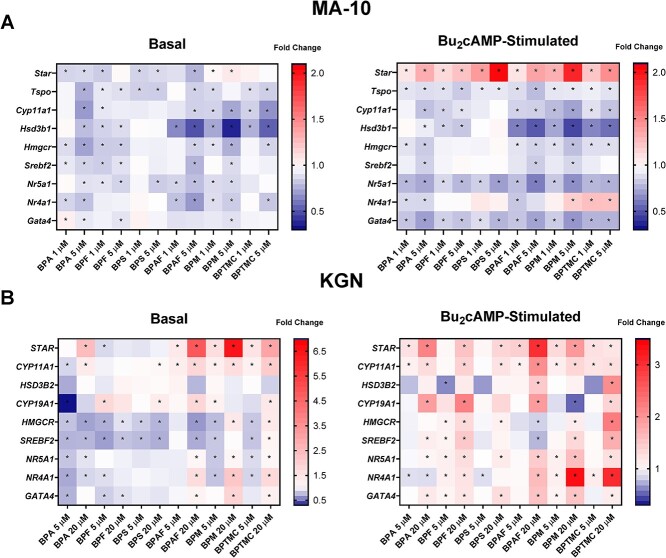
Heatmaps showing the mean fold change of gene expression in all transcripts tested. (A) MA-10 cells were treated with control, 1, or 5 μM of each bisphenol for 48 h, followed by a 2-h incubation with or without Bu_2_cAMP. (B) KGN cells were treated with control, 5, or 20 μM of each bisphenol for 48 h, in the absence or presence of Bu_2_cAMP. Results are displayed as a mean fold change, normalized to control. Two-way ANOVA with Dunnett test was performed. *P < 0.05, *N* = 5–7 (MA-10) and *N* = 5 (KGN).

In MA-10 cells, changes in the mRNA expression of *Srebf2* very closely paralleled those seen for *Hmgcr* (Figure 5C). There were no significant effects on *Srebf2* expression after treatment with BPS or BPTMC, under either basal or stimulated conditions, and BPF treatment under stimulated conditions had no effect. The other bisphenols caused a downregulation of expression. *SREBF2* expression also closely paralleled *HMGCR* expression in KGN cells (Figure 5D). Bisphenol TMC greatly increased *SREBF2* gene expression by 79% when compared to its stimulated control. The heatmaps in Figure 6 show the mean fold changes in all of the transcripts, summarizing the qRT-PCR data.

## Discussion

MA-10 Leydig and KGN granulosa cell lines are widely used as models to study testicular and ovarian steroidogenesis, respectively. MA-10 cells express functional luteinizing hormone receptors and steroid production is increased in response to exogenous stimulation with cAMP [32, 40, 41]. Like human granulosa cells, KGN cells have the ability to produce both progesterone and estradiol and have functional FSH receptors [33, 42]. KGN cells respond to FSH and hormone secretion is further increased after cAMP stimulation [33, 42]. Thus, we investigated the impact of BPA and five structural analogs on steroidogenesis using these representative in vitro models. Despite the widespread use of structural analogs of BPA in consumer products, relatively little is known about how exposure to these chemicals may impact the process of steroidogenesis. The cytotoxicity of the bisphenols was cell-line specific (Supplementary Figure S1), as reported in a previous study on MA-10 and KGN cells from our group [17].

Other studies have assessed the effects of structural bisphenol analogs on steroidogenesis in various cell types. Roelofs et al. reported that exposure to 10 μM BPF or BPS increased basal P4 secretion in MA-10 cells after 48 h [25]. In contrast to this observation, we did not observe a significant increase in basal P4 production after the exposure of MA-10 cells to either of these bisphenols. In mLTC-1 cells, exposure to 50 and 70 μM BPAF significantly decreased hCG-stimulated P4 production after 24 h [43]. Our data reveal that lower concentrations of BPAF increased both basal and Bu_2_cAMP-stimulated progesterone production in MA-10 cells.

In KGN cells, in contrast to our reported increase in basal P4 production after BPA exposure, another study observed that P4 was decreased when these cells were exposed to 0.5–500 μg/L BPA for 6 h [44]. However, in that study, the cells were exposed for only 6 h, whereas we exposed KGN cells to BPA for 48 h. We also found that Bu_2_cAMP stimulation reduced P4 production after exposure to all of the bisphenols with the exception of BPF. This is consistent with the report by Qi et al., in which exposure to 10 μM BPA decreased P4 levels in KGN cells in the presence of forskolin, another steroidogenic stimulant [45]. Together, these data indicate that the effects of bisphenols on steroid production are dependent on the cell line, the length of treatment, and the type of steroidogenic stimulus used.

Our data on E2 were also consistent with the study by Liu et al., in which low doses of BPA stimulated both *CYP19A1* expression and E2 secretion in KGN cells [46]. Another report investigating the effects of BPS on estrogen production found that BPS caused excessive estrogen synthesis in SVOG cells, another human ovarian granulosa cell line [47]. Both BPA and BPS also increased E2 secretion in ovine granulosa cells [48]. Interestingly, the levels of E2 measured in this study correlated well with *CYP19A1* gene expression. *CYP19A1* is involved in estrogen biosynthesis by transforming androstenedione to estrone (which can then be converted to estradiol) and testosterone to estradiol. We found that exposure to 5 or 20 μM BPF, BPS, BPAF, BPM, and BPTMC led to an increase in basal *CYP19A1* expression. Exposure to 5 or 20 μM BPA, BPF, BPS, BPAF, and BPTMC also led to an increase in Bu_2_cAMP-stimulated *CYP19A1* gene expression; 20 μM BPM downregulated *CYP19A1*. Gene expression data for this transcript correlated well with the levels of estradiol measured in cells. Indeed, exposure to 5 or 20 μM BPA, BPF, BPS, BPAF, and BPTMC upregulated basal E2 levels while exposure to 5 or 20 μM BPA, BPF, BPS, and BPAF increased Bu_2_cAMP-stimulated E2 production. After stimulation, BPM and BPTMC decreased E2 secretion.

To elucidate the basis for the effects on steroidogenesis, we investigated the impact of bisphenols on the expression of genes directly involved in the steroidogenic pathway or responsible for cholesterol transport and biosynthesis. STAR is crucial for transporting cholesterol from the outer to the inner mitochondrial membrane, where cholesterol can then serve as the substrate for sex hormone production [49, 50]. Translocator protein has been proposed to interact with STAR during the translocation of cholesterol [51]. Interestingly, the importance of TSPO in steroidogenesis has been debated. The observation that TSPO-knockout MA-10 cells produced the same amount of P4 as control cells after Bu_2_cAMP treatment suggested that the absence of *Tspo* may not have a critical impact on P4 production [52]. However, Fan et al. showed that a mutation in the *Tspo* gene in MA-10 cells greatly reduced P4 production in response to Bu_2_cAMP, altered lipid homeostasis, and induced *Star* expression [53]. Further research is needed to resolve the role of TSPO in KGN cells. To the best of our knowledge, this is the first study to assess the effects of exposure to various bisphenols on the expression of *Tspo* and *TSPO* in MA-10 and KGN cells, respectively.

In the inner mitochondrial membrane, cholesterol is converted to pregnenolone by CYP11A1, which is subsequently metabolized into progesterone by HSD3B [49, 50]. The effects of exposure to bisphenols on the expression of these transcripts were both bisphenol and cell line specific. A variety of effects have also been reported in the literature. For example, in BLTK-1 murine Leydig cells, progesterone secretion was not affected by BPA, but exposure to 0.01 or 0.1 μM BPA downregulated the expression of *Star* and *Cyp11a1* [54]. Previously, Qi et al. reported that, in the presence of forskolin, the exposure of KGN cells to 10 μM BPA increased mRNA levels of *STAR* and had no effect on *HSD3B2*. In the same study, BPA had no effect on *CYP11A1*; however, the cells were only treated for 24 h [45]. Similarly, other bisphenol compounds have been reported to disrupt steroidogenesis. In granulosa cells isolated from women undergoing IVF, exposure to 10 or 50 μM BPS for 48 h decreased basal progesterone secretion but had no effect on *STAR* gene expression [55]. Exposure to 100 μM BPA or BPF for 72 h decreased basal progesterone secretion and exposure to 100 μM BPAF, BPF, BPS, or BPA decreased FSH-stimulated progesterone levels in porcine ovarian granulosa cells [56]. Interestingly, in that study no changes were observed in the gene expression levels of *STAR*, *CYP11A1*, *HSD3B*, or *CYP19A1* [56].

The discordance between the modulation of gene expression and alterations in hormone secretion observed in both MA-10 and KGN cells may be due to other, lesser known mechanisms of action of the bisphenols [57]. Indeed, BPA has been shown to activate the steroid and xenobiotic receptor/pregnane X receptor (SXR/PXR) in humans, which may alter steroid hormone metabolism [57–59]. Furthermore, bisphenols may alter steroidogenesis not only by altering the expression of transcripts for key steroidogenic enzymes, but also by modulating post-transcriptional effects and/or the activity of these enzymes. Bisphenol A was found to inhibit the activities of HSD3B and CYP17A1 [60]. Thus, alterations in steroid hormone levels may occur in the absence of changes in transcript expression.

To determine whether some of the changes observed were due to the upstream regulators of the steroidogenic pathway, we measured the expression of transcripts for NR5A1, NR4A1, and GATA4. Previous studies have shown that BPA increased the expression of *Nr4a1* in K28 mouse testicular Leydig cells and decreased the expression of *NR5A1* and *GATA4* in human, IVF-derived, granulosa cells [61, 62]. Although these three upstream transcripts are important regulators of many steroidogenic enzymes, such as STAR, CYP11A1, and CYP19A, these enzymes are also regulated by other pathways and transcription factors [63]. In this study, we focused on the cAMP-dependent pathway of steroid production; however, there are cAMP-independent pathways that may also regulate steroid biosynthesis [64].

Cholesterol is a precursor for the synthesis of testosterone, estradiol, and progesterone [65, 66]. Thus, it was of interest to examine whether the bisphenols tested affected the expression of transcripts involved in the de novo synthesis of cholesterol. Our results show that exposure to several bisphenols downregulated *Hmgcr* and *Srebf2* expression in MA-10 cells, under both basal and stimulated conditions. In KGN cells, some bisphenols induced a downregulation while others upregulated *HMGCR* and *SREBF2* expression. Thus, bisphenols may modulate the levels of cholesterol within the cells by affecting the transcripts involved in cholesterol biosynthesis. However, the de novo pathway is not the only way that MA-10 and KGN cells can acquire cholesterol for steroidogenesis. In Leydig and granulosa cells, cholesterol may be obtained from the plasma membrane or through lipid droplets; these droplets act as storage for cholesteryl esters [49, 67, 68]. After de-esterification, the cholesterol released from lipid droplets may be used as a substrate for steroidogenesis [49]. Previous work from our group has shown that exposure of MA-10 cells to 5 and 10 μM BPTMC decreased lipid droplet areas and numbers, while exposure of KGN cells to 100 μM BPA or BPF, or 20 μM BPM resulted in an increase in lipid droplet numbers [17]. It remains unknown whether these droplets are rich in cholesteryl esters [17]. Therefore, a better understanding of the composition of these lipid droplets will be required to establish how a change in their areas or numbers impacts on steroidogenesis in MA-10 and KGN cells.

Exposure of KGN cells to BPA increased the expression of ATP binding cassette subfamily A member 1 (ABCA1) [45]. ATP binding cassette subfamily A member 1 mediates the export of cholesterol; an increase in the mRNA and protein levels of ABCA1 may decrease the intracellular pools of cholesterol that may provide an explanation for the reduction of progesterone observed after exposure to BPA [45]. In humans, exposure to BPA was associated with increased serum levels of LDL cholesterol (LDL-C), an increased total cholesterol (TC) to HDL cholesterol (HDL-C) ratio, and decreased levels of HDL-C and triglycerides (TG) [69]. In C57BL/6 mice, exposure to BPA increased serum TC and LDL-C, decreased serum HDL-C, and upregulated the mRNA and protein expression levels of HMGCR and SREBP2 [70]. In mice fed a high-fat diet, exposure to BPA, BPF, or BPAF altered fatty acid composition, and BPF or BPAF lowered glyceride and cholesterol levels [71]. In male rats, exposure to BPAF decreased serum TC and the mRNA expression levels of SREBP-1c and SR-B1, a protein involved in the transport of cholesterol [72]. Lastly, exposure to BPS increased serum TC and TG in male Sprague–Dawley rats [73]. It is clear that BPA and several of its analogs are altering lipid homeostasis. However, it remains unclear whether and how the effects of the bisphenols on cholesterol homeostasis and the de novo cholesterol synthesis pathway contribute directly to their effects on progesterone or estradiol production in MA-10 and KGN cells. Further research will also be necessary in order to determine how disruptions in the synthesis of these steroid hormones may ultimately contribute to adverse effects on male and female reproduction.

In conclusion, we observed clear cell line, chemical, and concentration-dependent effects (Figure 6). Many of the effects also differed based on the presence or absence of Bu_2_cAMP stimulation. In MA-10 cells, there was an overall inhibition in the steroidogenic and cholesterol biosynthesis pathways under both the basal and stimulated conditions; after stimulation, *Star*, the exception to this observation, was consistently upregulated by the bisphenols. In KGN cells, effects on transcripts were more varied under basal conditions; under stimulated conditions, there was an overall stimulation in the steroidogenic and cholesterol biosynthesis pathways. In both cell lines, BPS had relatively fewer effects on the expression of the transcripts compared to the other five bisphenols. To the best of our knowledge, this is the first study to demonstrate that five structural analogs of BPA can disrupt steroid production in two steroidogenic cell lines and alter the expression of transcripts involved in this process. This study is the first to provide insight into the effects of two less studied BPA analogs, BPM and BPTMC. Our data indicate that certain BPA replacements have the potential to have greater adverse effects than BPA. This knowledge will not only help to inform future decision making about emerging chemicals prior to their adoption as substitutes for BPA, but will also aid in identifying responsible replacements.

## Supplementary Material

Supplemental_File_Iskandarani_et_al_November2024_ioae165

## Data Availability

Data are available on request.
