# Supplementary material for: The effects of bisphenol A and its analogs on steroidogenesis in MA-10 Leydig cells and KGN granulosa cells
Source: Biol Reprod. 2024 Nov 9;112(2):399–414. doi: 10.1093/biolre/ioae165 (PMC11833478; doi:10.1093/biolre/ioae165)

**Supplemental Figure S1.** Effects of bisphenols on cell numbers in (A) MA-10 and (B) KGN cells. Cells were treated with increasing concentrations of six different bisphenols for 48 h, and the nuclei were then stained with Hoechst 33342 and analyzed by high-content imaging. Cytotoxicity is defined as less than 70% viable cells (dotted line). Results are shown as mean % of control ± SEM. Holm-Bonferroni corrected one sample t-tests were performed. **P* < 0.05, ***P* < 0.01, ****P* < 0.001 compared to control, N = 8 (MA-10) and N = 6 (KGN).


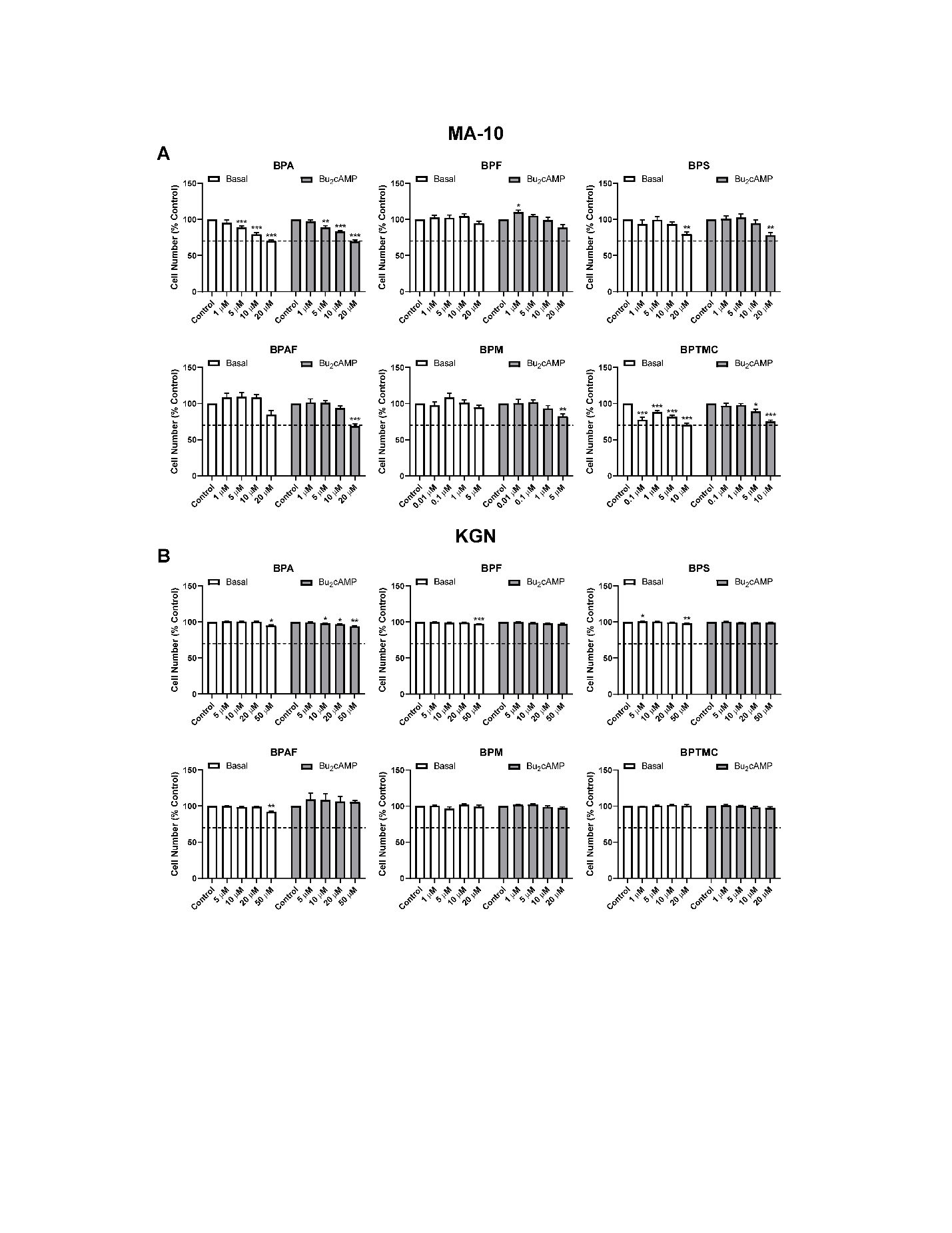


**Supplemental Figure S2.** Heatmaps showing the mean fold change of progesterone and estradiol production. (A) MA-10 cells were treated for 48 h, followed by a 2-h incubation with or without Bu2cAMP. (B) KGN cells were treated for 48 h in the absence or presence of Bu_2_cAMP. Supernatant was collected and progesterone (P4) or estradiol (E2) were measured in the medium. To measure the production of E2, the cells were additionally supplemented with 10 μM androstenedione. All cells were stained with Hoechst 33342 and counted by high-content imaging for normalization. Red signifies an upregulation and blue signifies a downregulation in P4 or E2 production. Results are displayed as a mean fold change normalized to control. Two-way repeated measures ANOVA followed by Dunnett’s test was performed. Boxes marked with an X indicate that a bisphenol has not been tested at that concentration; **P* < 0.05, N = 8 (MA-10) and N = 6 (KGN).

**
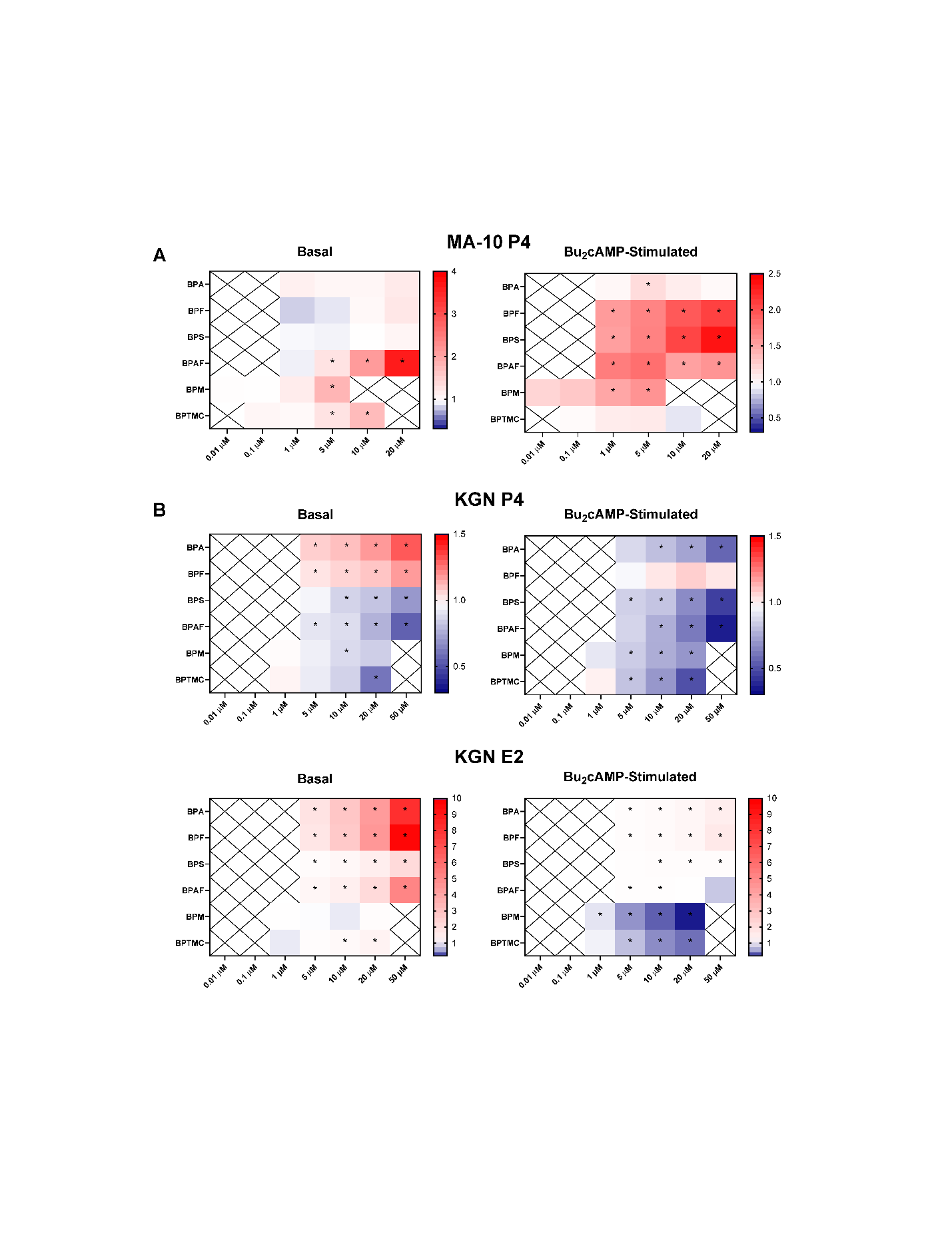
**

**Supplemental Figure S3.** Effects of bisphenols on the translocator protein and aromatase transcripts. MA-10 cells (left) were treated for 48 h with control, 1, or 5 µM of each bisphenol, followed by a 2-h incubation with or without Bu2cAMP. KGN cells (right) were treated for 48 h with control, 5, or 20 µM of each bisphenol, in the absence or presence of Bu_2_cAMP. Bar graphs show the effects of the six bisphenols on the relative expression of (A) *Tspo*, (B) *TSPO*, and (C) *CYP19A1*. Data represent means ± 95% CI. qRT-PCR expression was normalized to *Hprt* (MA-10) or *GAPDH* (KGN). Two-way ANOVA with Dunnett’s test was performed; **P* < 0.05 compared to basal control, #*P* < 0.05 compared to stimulated control, N = 6–7 (MA-10) and N = 5 (KGN).


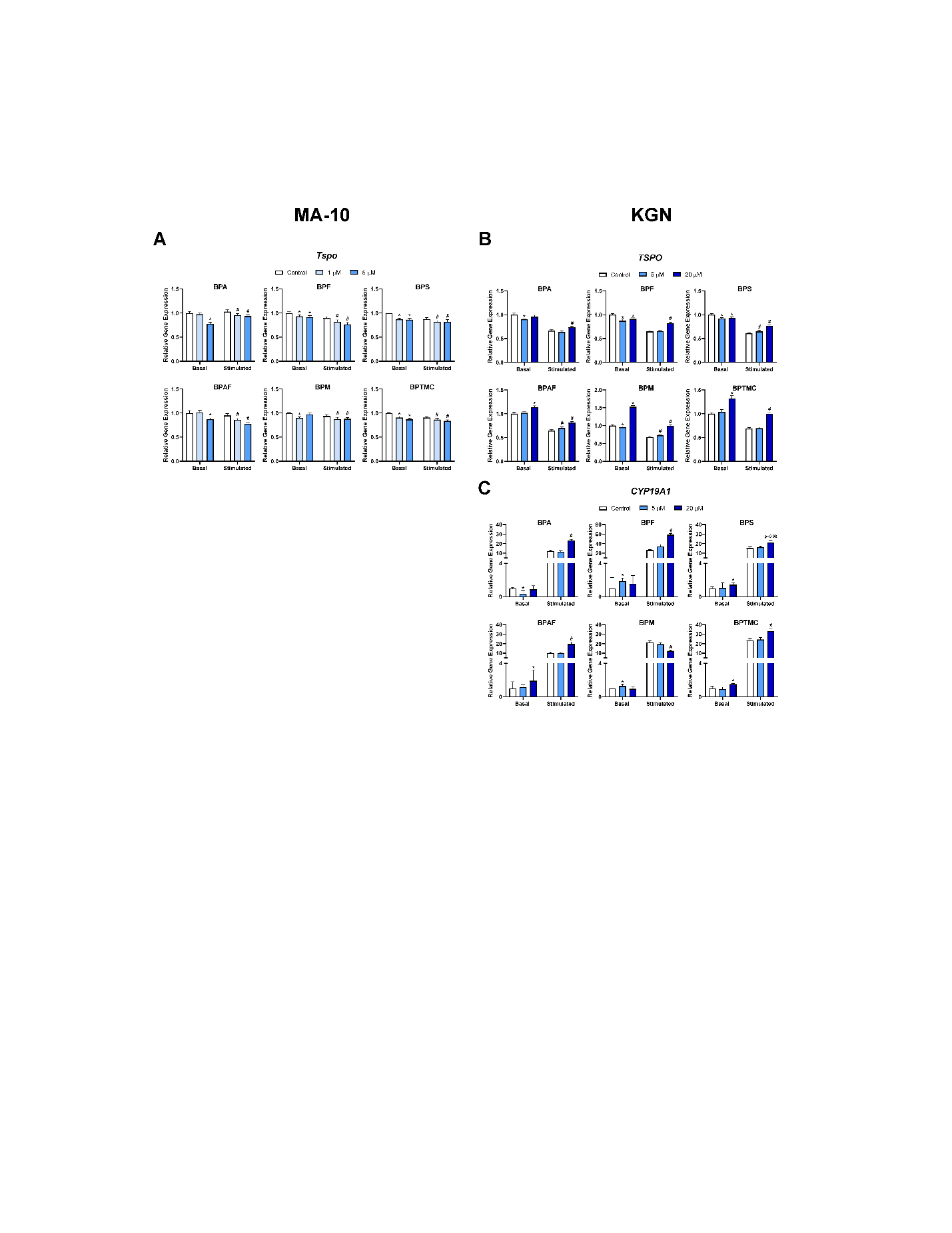

Supplement: Supplemental_File_Iskandarani_et_al_November2024_ioae165 [file supplemental_file_iskandarani_et_al_november2024_ioae165.docx]
